# Examination of the New ICD-11 Prolonged Grief Disorder Guidelines Across Five International Samples

**DOI:** 10.32872/cpe.4159

**Published:** 2021-03-10

**Authors:** Clare Killikelly, Mariia Merzhvynska, Ningning Zhou, Eva-Maria Stelzer, Philip Hyland, Jose Rocha, Menachem Ben-Ezra, Andreas Maercker

**Affiliations:** a Department of Psychology, University of Zurich, Zurich, Switzerland; b Department of Psychology and Cognitive Science, East China Normal University, Shanghai, China; c Department of Psychology, University of Arizona, Tucson, AZ, USA; d Department of Psychology, Maynooth University, Maynooth, Ireland; e Instituto Universitário de Ciências da Saúde, Gandra, Portugal; f School of Social Work, Ariel University, Ariel, Israel; Philipps-University of Marburg, Marburg, Germany

**Keywords:** prolonged grief disorder, ICD-11, psychometric validity, global applicability

## Abstract

**Background:**

Prolonged grief disorder (PGD) is a new disorder included in the 11th edition of the International classification of diseases (ICD-11). An important remit of the new ICD-11 is the global applicability of the mental health disorder guidelines or definitions. Although previous definitions and descriptions of disordered grief have been assessed worldwide, this new definition has not yet been systematically validated.

**Method:**

Here we assess the validity and applicability of core items of the ICD-11 PGD across five international samples of bereaved persons from Switzerland (N = 214), China (N = 325); Israel (N = 544), Portugal (N = 218) and Ireland (N = 830).

**Results:**

The results confirm that variation in the diagnostic algorithm for PGD can greatly impact the rates of disorder within and between international samples. Different predictors of PGD severity may be related to sample differences. Finally, a threshold for diagnosis of clinically relevant PGD symptoms using a new scale, the International Prolonged Grief Disorder Scale (IPGDS), in three samples was confirmed.

**Conclusions:**

Although this study was limited by lack of questionnaire data points across all five samples, the findings for the diagnostic threshold and algorithm iterations have implications for clinical use of the new ICD-11 PGD criteria worldwide.

## Abstract (highlights)

- The first study to explore core items of the ICD-11 PGD definition in five large international samples
- Comparison of three different diagnostic algorithms
- Preliminary analysis of different thresholds for diagnosis in different groups
- Preliminary estimates of PGD prevalence

In 2019 prolonged grief disorder (PGD) was included in the International Classification of Diseases (ICD-11) for the first time. The diagnostic criteria for a disorder of grief have a long history and there are several previous definitions and iterations (Prigerson et al., 2009; Shear, 2015; Wagner & Maercker, 2010). The current definition represents a new focus of the World Health Organization (WHO) on the clinical utility and global applicability of the disorder (Maercker et al., 2013). The rationale for the updated iteration in the new ICD-11 definition was to standardize this diagnosis internationally, however, the validity of the diagnostic criteria across different international samples has yet to be established. In this brief report, we test, for the first time, the core items of the PGD ICD-11 criteria in five international datasets.

The WHO working groups for the ICD-11 adopted a two-phase strategy to update disorder definitions. The first phase involved developing the structure of the definition based on a large international survey of psychologists and psychiatrists (Evans et al., 2013; Reed, Correia, Esparza, Saxena, & Maj, 2011). They called for flexible diagnostic guidelines, recognition of cultural factors, and fewer disorder categories with no subtypes. The resulting PGD definition included two core symptoms (intense yearning or preoccupation with the deceased), examples of emotional pain (i.e anger, sadness, guilt), at least 6 months duration since loss, and an impairment criterion. For a full description see Killikelly and Maercker (2017). Importantly, the working group also included a cultural caveat whereby symptoms of grief must exceed expected socio-cultural norms. The second phase in the WHO’s research approach was to evaluate the usability (clinical utility) of these guidelines in diagnostic decision making. Recent field studies have been conducted to explore the clinical utility and validity of PGD through clinicians’ assessments of vignettes (Keeley et al., 2016; Reed et al., 2018) and proposals for further evaluation (Gureje, Lewis-Fernandez, Hall, & Reed, 2019). These studies confirmed that, when compared with the ICD-10, the current ICD-11 including PGD improved the diagnostic sensitivity of grief related psychopathology, especially once the duration since loss criteria was included. However, until now this evaluative phase is limited and there are large scientific gaps in establishing the validity of the new ICD-11 PGD, particularly in a global context (Boelen, Spuij, & Lenferink, 2019; Eisma & Lenferink, 2018).

Previous research has confirmed that PGD may have different prevalence rates in different samples. For example, worldwide rates of a disorder of grief may range from 1% to 10% (Kristensen, Weisæth, & Heir, 2012; Lundorff, Holmgren, Zachariae, Farver-Vestergaard, & O’Connor, 2017). In a recent scoping review we found that the rates of disordered grief appear to be much higher in Asian countries compared to countries in Europe and North America (Stelzer, Zhou, Maercker, O’Connor, & Killikelly, 2020). This may depend on different factors including heterogeneity in the diagnostic criteria used, the sample characteristics, and, perhaps, specific cultural factors that may influence the assessment and reporting of grief symptoms. In this study, we sought to eliminate the methodological variability of previous studies by directly comparing some of the same diagnostic criteria items across multiple national samples, as well as exploring the sample characteristics and their influence on PGD symptoms.

This paper explores core items of the new ICD-11 PGD disorder criteria along with some of the supplementary items indicating emotional distress, across five international samples. The aims include: firstly, the examination of rates of possible PGD caseness using the same core items and diagnostic formulations in each country. Secondly, examination of criterion validity through the identification of predictors of PGD across and between countries. Thirdly, to find provisional cut-off scores and assess the thresholds for the best sensitivity and specificity in each country using the receiver operating characteristic analysis (ROC).

## Method

### Participants

Data from participants who experienced the loss of a loved one were analyzed. Data sets were obtained from five different countries: Switzerland (*N* = 214), China (*N* = 325), Israel (*N* = 544), Portugal (*N* = 218), and Ireland (*N* = 830). For demographic information see Table 1.1. For additional demographic characteristics for each sample please see Tables 1-4 in the Supplementary Materials.

### Recruitment and Sampling

Across all of the studies participants were recruited using online survey methods. In addition, the Portuguese data also includes a clinical outpatient sample. *Switzerland:* Data was collected using an online survey (Qualtrics). Participants were recruited through online and in person fliers posted at German speaking grief and bereavement support groups, online forums and community services (i.e. churches, townhalls, libraries). *China:* Participants were recruited to participate in an online survey (Qualtrics) using social media (WeChat) and online bereavement forums. *Israel:* Participants were recruited as part of a large national online survey using stratified and random sampling methods. *Ireland:* A nationally representative sample were recruited using the company Qualtrics. Stratified sampling methods were used to select participants based on sex, age and geographical location. *Portugal:* The ‘general’ group were recruited using Limesurvey anonymous online survey protocol using the snowball method. The ‘clinical group’ is based on participants from a Hospital setting (Centro Hospitalar Tâmega e Sousa) where participants received outpatient support for grief difficulties. Participants in this group were referred to the Grief Consultation Service part of the Clinical Psychology Unit and had completed informed consent procedures. This service is focused on supporting parental and perinatal losses and data was collected in face-to-face interviews with self-evaluation questionnaires.

### Measures

To assess prolonged grief disorder, the International Prolonged Grief Disorder Scale with 15 items (Killikelly et al., 2020) and the Inventory of Complicated Grief-Revised with 8 items (ICG-R; Prigerson et al., 2009; Prigerson & Jacobs, 2001) were used. Both instruments include two core PGD symptoms (i.e. yearning for the deceased and preoccupation), emotional distress symptoms as well as a measure of functional impairment, and time since loss. For the items of the IPGDS please see Killikelly et al. (2020). The following 8-items of the ICG-R were assessed: core items 1) ‘I think about him/her so much that it can be hard for me to do the things I normally do’ 2) ‘I feel myself longing and yearning for him/her’; accessory symptoms or examples of emotional distress, 3) ‘I feel as if a part of me died’ 4) ‘I feel disbelief over his/her death’ 5) ‘Ever since he/she died, I find it difficult to move on with my life’ 6) ‘I am bitter over his/her death’ 7) ‘I feel that it is unfair that I should live when he/she died’ and functional impairment criterion, 8) ‘I believe that my grief has resulted in impairment in my social, occupational or other areas of functioning. Unlike the ICG-R, the IPGDS includes one cultural item (i.e. My grief would be considered worse, e.g., more intense, severe and/or of longer duration, than for others from my community or culture). Participants were asked to rate their grief symptoms on a five-point scale (i.e. “not at all” on IPGDS or “almost never” on ICG-R (1), “rarely” (2), “sometimes” (3), “often” (4), “always” (5)). When filling out the IPGDS, participants were asked to mark the answer that best describes their feelings, thoughts and behaviour during the last week. In case of ICG-R, they were requested to select an answer that best describes how they felt during the last month. PGD was assessed using the IPGDS in Switzerland, China, and Portugal, and the ICG-R in all five countries. Recently the IPGDS was confirmed to be psychometrically reliable and valid with strong internal consistency (Cronbach's α = .92), high concurrent and criterion validity (see Killikelly et al., 2020). Previously the 8-item ICG-R was shown to have good reliability (Cronbach's α = .94) (Killikelly et al., 2019).

### Predictors

Life Events Checklist (LEC) (Gray, Litz, Hsu, & Lombardo, 2004) and International Trauma Exposure Measure (ITEM) (Hyland et al., 2020) items were measured on a binary scale (0 = no; 1 = yes). For the LEC response options 1-2 (happened to me, witnessed it) were merged into ‘yes’ while all other response options were merged into ‘no’. Information about traumatic events was not collected for the Portuguese sample. Furthermore, in the Portuguese sample, the duration since loss was not assessed and the data set revealed a high quantity of missing values (100 out of 218 participants) on the ICG-R scale. Therefore, the Portuguese sample was excluded from the data analysis when the association between predictors and PGD was investigated. The cultural item was collected only in Switzerland, China, and Portugal. The following variables were included in the data analysis as predictors of PGD:

1. Gender (measured in all 5 samples)
2. Age (measured in all 5 samples)
3. Cultural criteria (measured in Swiss, Chinese, Portuguese samples)
4. Severe human suffering (measured in Swiss, Chinese, Israeli samples with LEC, and in Irish sample with ITEM)
5. Sudden, violent or accidental death (measured in Swiss, Chinese, Israeli samples with LEC and in Irish sample with ITEM)
6. Serious injury, harm or death you caused to someone (measured in Swiss, Chinese, Israeli samples with LEC and in Irish sample with ITEM)

### Statistical Analysis

To estimate possible PGD rates, three different diagnostic algorithms were applied; PGD strict criteria set, PGD moderate criteria set, and the criteria set according to Maciejewski et al. (2016). PGD strict criteria set requires the endorsement of at least one core item, at least one item of emotional distress symptoms, and functional impairment; all of which are rated as 4 (often) or higher. PGD moderate criteria set has almost the same requirements except all items are rated 3 (sometimes) or higher (Killikelly et al., 2020). Criteria according to Maciejewski et al. includes at least one of two core items, three or more emotional distress items (all rated 4 (often) or above), and no functional impairment. In all three diagnostic algorithms the same time criterion was applied (i.e., loss occurred 6 months ago or longer). The estimated rates of possible PGD were calculated across the five samples with 95% Confidence Interval (CI). However, it is important to note that some key items were missing in the datasets. In the Portuguese and the Israeli samples the time criteria was not applied due to the absence of the data about time since loss and in the Portuguese dataset the functional impairment criterion was not evaluated. Therefore we can only examine estimates of possible PGD caseness not prevalence.

Logistic regression was used to examine the associations between PGD (strict criteria) and some items representing traumatic life events, gender (male/female), age, and cultural caveat item using odds ratio (OR) and 95% CI. The outcome was the endorsement of PGD strict criteria; coded as binary variable “yes, possible PGD caseness” (1) or “no” (2). Of note, due to the use of heterogeneous questionnaires across the samples, we could only include a few traumatic life event items. In terms of missing values, the default settings of SPSS were used whereby cases were deleted in a list wise manner. Third, Receiver operating characteristic analysis (ROC) was used to examine cut-off scores for the IPGDS and ICG-R, i.e. the threshold for the best fit in terms of sensitivity (high > .80) and specificity (.80). This analysis is presented as an initial exploration and may be highly dependent upon the samples used. ROC curves and logistic regression were calculated only for PGD strict criteria (i.e. 12 symptom items plus functional impairment). Statistical analyses were performed using SPSS version 23.

## Results

### Rates of PGD

The proportion of people in each sample who met the criteria for possible PGD caseness differed within the country depending on (1) whether strict, moderate or Maciejewski et al. (2016) diagnostic criteria were applied and (2) whether IPGDS or ICG-R were used to assess it. Furthermore, there was a difference in rates between the countries, even if assessed with the same diagnostic algorithm and the same measure instrument. For example using the strict criteria of the IPGDS the rates ranged from 6.9% to 12.6%, whereas for the ICG-R rates ranged from 2.0% to 21.1%. For detailed rates and confidence intervals (CI) see Table 1.1 and Table 1.2.

**Table 1.1:** Basic Sociodemographic Characteristics and Predictors in Five Samples

Variable	Swiss (*n* = 214) (*M*_Age_ = 38.7)		Chinese (*n *= 325) (*M*_Age_ = 33.3)		Israel (*n *= 544) (*M*_Age_ = 41.4)		Portuguese (*n *= 218) (*M*_Age_ = 32.8)		Irish (*n *= 830) (*M*_Age_ = 45.4)	
	***n***	**%**	***n***	**%**	***n***	**%**	***n***	**%**	***n***	**%**
Gender										
Male	33	15.4	104	32	246	45.2	43	17.5	411	49.5
Female	178	83.2	212	65.2	298	54.8	203	82.5	419	50.5
Other	3		2		0		0		0	
Item										
Severe human suffering (LEC Item 13)	83	38.8	65	20.0	39	7.1	–	–	–	–
Sudden, violent death (LEC Item 14)^a^	62	29.0	53	16.3	71	13.0	–	–	–	–
Accidental death (LEC Item 15)	57	26.6	99	30.5	173	31.8	–	–	–	–
Serious injury, harm or death you caused (LEC Item 16)	6	2.8	49	15.1	11	2.0	–	–	–	–
Serious injury, harm or death you caused (ITEM Item 12)	–	–	–	–	–	–	–	–	35	4.2
Sudden, violent or accidental death (ITEM Item 13)	–	–	–	–	–	–	–	–	224	27.0

^a^LEC items 14 and 15 were merged in the logistic regression. Data was not collected for the Portuguese sample.

**Table 1.2:** Estimates of Possible PGD Using Different Diagnostic Rules Across Five Countries

Scale	Swiss^ ^ (*n* = 214)			China^ ^ (*n* = 325)			Israel^a^ (*n* = 544)			Portuguese^b^ (*n* = 218)			Irish^ ^ (*n* = 830)		
	%	95% CI		%	95% CI		%	95% CI		%	95% CI		%	95% CI	
		*LL*	*UL*		*LL*	*UL*		*LL*	*UL*		*LL*	*UL*		*LL*	*UL*
IPGDS															
Strict criteria	7.0	4.0	11.3	12.6	9.2	16.7	–	–	–	6.9	3.9	11.1	–	–	–
Moderate criteria	21.5	16.2	27.6	37.5	32.3	43.1	–	–	–	27.5	21.7	34.0	–	–	–
Maciejewski criteria	15.9	11.3	21.5	33.5	28.4	39.0	–	–	–	23.4	17.9	29.6	–	–	–
ICG-R										**(*n* = 118)** *Estimate only*					
Strict criteria	5.1	2.6	9.0	7.1	4.5	10.4	2.0	1.0	3.6	21.1	14.2	29.7	4.1	2.9	5.7
Moderate criteria	18.2	13.3	24.1	29.2	24.3	34.5	8.5	6.3	11.1	48.3	39.0	57.7	13.9	11.6	16.4
Maciejewski criteria	6.1	3.3	10.2	10.5	7.4	14.3	4.2	2.7	6.3	7.6	3.5	14.0	4.7	3.4	6.4

^a^In Israel dataset for ICG-R – no time criteria applied.

^b^In Portuguese dataset for ICG-R – no time criteria applied, no functional criteria (Item 8) applied; for IPGDS - no time criteria applied, pooled across the general and clinical groups.

### Logistic Regression

Results from the logistic regression analyses showed that PGD assessed with IPGDS was significantly associated with the cultural caveat criteria in Switzerland, *OR* = 2.463, 95% CI [1.707, 3.554], and in China, *OR* = 3.152, 95% CI [2.361, 4.209]; with serious injury, harm or death to someone else, *OR* = 14.016, 95% CI [1.856, 105.854], in Switzerland, and with gender (higher risk for women), *OR* = 0.508, 95% CI [0.259, 0.998] in China (see Table 2.1).

**Table 2.1:** Logistic Regressions for a Set of Predictor Variables Associated With PGD Measured With IPGDS

Variable	Swiss (*n* = 201)			China (*n* = 302)		
	*OR*	95% CI		*OR*	95% CI	
		*LL*	*UL*		*LL*	*UL*
IPGDS						
Gender^a^	1.240	0.331	4.646	0.508*	0.259	0.998
Age	1.018	0.989	1.049	1.022	0.996	1.048
Cultural criteria	2.463***	1.707	3.554	3.152***	2.361	4.209
Severe human suffering	2.321	0.898	6.000	1.256	0.507	3.111
Sudden, violent or accidental death	1.821	0.734	4.517	0.703	0.342	1.448
Serious injury, harm or death you caused	14.016*	1.856	105.854	1.471	0.534	4.055

^a^Female compared to male.

*p < .05. **p < .01. ***p < .001.

When PGD was assessed with ICG-R, the logistic regression analyses revealed significant associations with the cultural caveat criteria within Switzerland, *OR* = 8.148, 95% CI [2.629, 24.782], and China, *OR* = 4.501, 95% CI [2.671, 7.586]; with serious injury, harm or death person caused to someone in China, *OR* = 5.494, 95% CI [1.309, 23.050]; with age, *OR* = 0.964, 95% CI [0.933, 0.966], severe human suffering, *OR* = 5.095, 95% CI 1.670, 15.547], and with sudden, violent or accidental death, *OR* = 3.271, 95% CI [1.178, 9.086], in Israel, and finally with gender, *OR* = 0.993, 95% CI [0.967, 1.020], and sudden, violent or accidental death, *OR* = 0.297, 95% CI [0.127, 0.694], in Ireland (see Table 2.2).

### Examination of Provisional Cut-Off Scores

The ROC analysis was used to determine a cut-off score for those participants meeting the strict criteria for the IPGDS and ICG-R. The results can be found in Table 3. The Chinese sample required a slightly higher cut-off score (42.5) for the IPGDS when compared to the Swiss (37.5) and Portuguese (36.5) samples. Additionally, for the ICG-R the Portuguese sample had a lower cut-off (16.5) when compared with the Swiss (24.5), Chinese (25.5), Israeli (24.5) and Irish (22.5) samples.

**Table 2.2:** Logistic Regressions for a Set of Predictor Variables Associated With PGD as Measured by ICG-R

Variable	Swiss (*n* = 201)			China (*n* = 302)			Israel (*n* = 544)			Irish (*n* = 830)		
	*OR*	95% CI		95% CI			*OR*	95% CI		*OR*	95% CI	
		*LL*	*UL*	*OR*	*LL*	*UL*		*LL*	*UL*		*LL*	*UL*
ICG-R												
Gender^a^	1.319	0.109	15.984	0.407	0.139	1.192	0.847	0.347	2.068	0.303**	0.967	1.020
Age	1.060	1.000	1.124	1.023	0.984	1.063	0.964*	0.933	0.966	0.993	0.133	0.692
Cultural criteria	8.148***	2.629	24.782	4.501***	2.671	7.586	–	–	–	–	–	–
Severe human suffering	1.495	0.290	7.708	0.286	0.057	1.428	5.095**	1.670	15.547	0.535	0.249	1.149
Sudden, violent or accidental death	0.779	0.147	4.117	0.809	0.247	2.648	3.271*	1.178	9.086	0.297**	0.127	0.694
Serious injury, harm or death you caused	19.536	0.266	1433.830	5.494*	1.309	23.050	0.964	0.079	11.748	0.339	0.102	1.131

^a^Female compared to male.

*p < .05. **p < .01. ***p < .001.

**Table 3:** Receiver Operating Characteristic Analysis (ROC)

Scale	Swiss (*n* = 214)		China (*n* = 325)		Israel (*n* = 544)		Portuguese (*n* = 218)		Irish (*n* = 830)	
	cut-off [min; max]	sensitivity/ specificity	cut-off [min; max]	sensitivity/ specificity	cut-off [min; max]	sensitivity/ specificity	cut-off [min; max]	sensitivity/ specificity	cut-off [min; max]	sensitivity/ specificity
IPGDS	37.5 [13; 63]	0.933/ 0.814	42.5 [13; 65]	0.902/ 0.810	N/A	N/A	36.5 [13; 56]	0.933/ 0.818	N/A	N/A
ICG-R	24.5 [8; 40]	0.818/ 0.857	25.5 [8; 40]	0.957/ 0.854	24.5 [8; 40]	1.000/ 0.947	16.5 [7; 35]	0.920/ 0.871	22.5 [8; 40]	0.941/ 0.896

## Discussion

This paper provides the first systematic exploration of core items of the new ICD-11 PGD criteria across five international samples. The results confirm large differences in the rates between and within samples depending on the diagnostic algorithm used; predictors of PGD severity may vary across samples due to the type of loss (violent or nonviolent) and the cultural caveat item of the IPGDS may be an important risk screening item; finally, a threshold for a clinically relevant diagnosis may be different depending on cultural group.

Core items of the new ICD-11 PGD criteria, as tested by the IPGDS (in Swiss, Chinese and Portuguese samples) and the ICG-R (in Irish and Israeli samples), revealed substantially different rates depending on the diagnostic algorithm used. Overall, the *strict criteri*a for both the IPGDS and the ICG-R seems to capture the expected rates across the five samples, which ranged from 2-21.2%. However, substantially higher rates were found in the Chinese and Portuguese samples. There could be several explanations for these higher rates including sample differences and lack of cultural sensitivity of assessment measures (Stelzer, Zhou, & Maercker, et al., 2020). When the strict criteria of the IPGDS were applied, the Swiss (7.0%) and Portuguese (6.9%) samples had similar rates on the IPGDS, whereas the Chinese sample had a higher rate (12.6%) on the IPGDS. A higher rate in the Chinese sample is consistently found across all iterations of the IPGDS but also for most of the ICG-R comparisons. Conversely, when assessing the ICG-R the Swiss, Chinese, Israeli and Irish samples had similar rates, whereas the Portuguese sample was much higher (21.1%). The Portuguese sample also had high rates on the ICG-R for the strict and moderate criteria, perhaps due to the exclusion of the impairment criteria in this particular sample. Therefore, the results for the Portuguese sample must be interpreted with caution and it points to the importance of including the functional impairment item and ensuring consistency in the use of time criterion in the assessment measure. Additionally, the Portuguese sample included pooled data from the general and clinical sample. The inclusion of the clinical sample could increase the prevalence rates in the Portuguese data compared to the non-clinical samples obtained from the other countries.

The Portuguese sample consisted of a large proportion of bereaved people who experienced an unexpected loss (10%). Although not explicitly recorded, this would mostly include the unexpected loss of a child as participants were from the outpatient perinatal loss clinic. Loss of a child is known to predict high levels of PGD (Zetumer et al., 2015)

Lack of culturally sensitive assessment measures or items could explain differences in the symptom ratings and severity levels across the samples. For example, our previous study confirmed that Chinese bereaved may present with slightly different symptoms than those assessed by the ICD-11 (Killikelly & Maercker, 2017; Stelzer, Zhou, Merzhvynska, et al., 2020). The IPGDS standard scale does not explore somatic symptoms or culturally specific symptoms such as ‘a loss of a part of oneself’ (Stelzer, Zhou, Merzhvynska, et al., 2020). Additionally, there could be a cultural bias in responding to these questionnaires which may lead to overreporting and overestimation of symptoms. Chentsova-Dutton et al. (2007) found that Chinese participants may overreport certain symptoms in order to ensure that they receive health care and support.

In terms of predictors of PGD severity we assessed a limited selection of predictors available across the datasets. Interestingly, when the cultural caveat item was included (e.g. endorsement of Item 14 of the IPGDs), violating the cultural norms for grief was found to significantly predict more severe grief scores on the IPGDS and the ICG-R. Although we only had the data for the Swiss and Chinese participants, further examination of this item might indicate its importance as a screening item for grief severity. In both the Israeli and Irish sample grief severity was predicted by sudden violent or accidental death whereas this was not found for the Swiss and Chinese samples. This may be due to differences in sampling. The Israeli and Irish data are from large nationally representative samples that may include more instances of sudden violent or accidental death. The Chinese and Swiss samples are mostly student populations who experienced the loss of older relatives. The larger Israeli and Irish datasets contain participants who experienced a high level of violent loss (more than 25%) and this could explain the differences in predictors. Previous research has confirmed that violent loss is a strong predictor of PGD severity and chronicity (Lobb et al., 2010; Schaal, Jacob, Dusingizemungu, & Elbert, 2010). Additionally, Israel and Ireland have recently experienced acts of terrorism that may preclude an added cultural vulnerability to trauma and loss (Duffy, Gillespie, & Clark, 2007; Silverman, Johnson, & Prigerson, 2001).

The final research question was to determine a possible threshold for establishing a clinically significant severity score on the IPGDS. All five datasets could not be compared with the IPGDS however across the Swiss, Chinese and Portuguese data, a score above 36.5 will most likely represent clinically significant PGD symptoms. As a control, the ICG-R was also examined and a score above 22 for all datasets was consistently found, except for the Portuguese sample (16.5). This attests to the variation that can occur across different samples, even with gold standard clinical assessments (Boelen & Lenferink, 2020).

### Limitations

Due to inconsistencies in data collection across the five international samples it was not possible to directly compare the IPGDS or the ICG-R across all data sets. The full ICD-11 PGD criteria could therefore not be assessed. In particular the time criterion was not assessed consistently across the datasets for example not in the Portuguese or Israeli datasets. Therefore, a diagnosis of PGD is not possible. However, the core items of the PGD (yearning and preoccupation) as well as some supplementary items of emotional distress could be evaluated and indications of possible caseness implied. It is important to include the time criterion for disorder as individuals may experience severe distress in the first weeks and months after a loss and this should not be pathologized. Importantly the estimates of prevalence rates for the Portuguese data must be interpreted with caution as there was a high amount of missing data. Furthermore, the Portuguese sample included a clinical subgroup. This may explain why the estimates of prevalence are significantly higher. Across the German, Portuguese and Chinese samples there is a high proportion of female responses. In the future it would be important to provide an analysis of a more representative sample. Additionally, there were only a limited number of similar predictors across all datasets. The data in each country was collected separately at different times, so only a cross sectional comparison is possible on some questionnaire items. Of note, the confidence intervals are very wide for some of the items in the logistic regression, particularly for the cultural criteria. This is perhaps due to a small number of values in some of the cells (response options). In the future a larger sample size should reveal more precise confidence intervals. Finally, in the future and with a more complete dataset the ROC analysis should also be conducted on the moderate and Maciejewski et al. (2016) criteria to provide a full estimate of possible thresholds for sensitivity and specificity.

### Conclusion

This paper confirms the importance of establishing international guidance on the consistent use of a diagnostic algorithm for PGD in order to ensure reliability across heterogeneous samples. Currently, we recommend the use of the strict criteria as an indicator of PGD caseness, however this must be confirmed in a clinical sample. Future studies should examine the different PGD algorithms (moderate vs strict) in clinical and cultural samples and include important items that are missing in some of the current data (i.e. the impairment and time criteria as well as the cultural caveat). Additionally, clinicians should be aware of specific risk factors such as violent, sudden loss or screening ‘yes’ on the cultural caveat IPGDS item as these may predict clinically severe grief. In the future it may be important for clinicians to note that different cultural groups may need different cut-off thresholds for a clinical diagnosis on the IPGDS or other scales.

## Supplementary Materials

The supplementary information contains tables of additional demographic characteristics for each of the five samples (for access see Index of Supplementary Materials below).

10.23668/psycharchives.4159 Supplement 1 Supplementary materials to "Examination of the new ICD-11 prolonged grief disorder guidelines across five international samples" [Additional information]

KillikellyC.
MerzhvynskaM.
ZhouN.
StelzerE.-M.
HylandP.
RochaJ.
Ben-EzraM.
MaerckerA.
 (2021). Supplementary materials to "Examination of the new ICD-11 prolonged grief disorder guidelines across five international samples"
[Additional information]. PsychOpen. 10.23668/psycharchives.####PMC966712336397782

## References

[sp1_r1] Killikelly C. Merzhvynska M. Zhou N. Stelzer E.-M. Hyland P. Rocha J. Ben-Ezra M. Maercker A. (2021). Supplementary materials to "Examination of the new ICD-11 prolonged grief disorder guidelines across five international samples" [Additional information]. PsychOpen. 10.23668/psycharchives.#### PMC9667123 36397782

